# Carnosol, a Natural Polyphenol, Inhibits Migration, Metastasis, and Tumor Growth of Breast Cancer via a ROS-Dependent Proteasome Degradation of STAT3

**DOI:** 10.3389/fonc.2019.00743

**Published:** 2019-08-08

**Authors:** Halima Alsamri, Hussain El Hasasna, Yusra Al Dhaheri, Ali H. Eid, Samir Attoub, Rabah Iratni

**Affiliations:** ^1^Department of Biology, College of Science, United Arab Emirates University, Al Ain, United Arab Emirates; ^2^Department of Pharmacology and Toxicology, Faculty of Medicine, American University of Beirut, Beirut, Lebanon; ^3^Department of Pharmacology and Therapeutics, College of Medicine and Health Sciences, United Arab Emirates University, Al-Ain, United Arab Emirates

**Keywords:** triple negative breast cancer (TNBC), stat3, reactive oxygen species, proteasome, tumor growth, metastasis

## Abstract

We have previously demonstrated that carnosol, a naturally occurring diterpene, inhibited *in vitro* cell viability and colony growth, as well as induced cell cycle arrest, autophagy and apoptosis in human triple negative breast cancer (TNBC) cells. In the present study, we evaluated the ability of carnosol to inhibit tumor growth and metastasis *in vivo*. We found that non-cytotoxic concentrations of carnosol inhibited the migration and invasion of MDA-MB-231 cells in wound healing and matrigel invasion assays. Furthermore, gelatin zymography, ELISA, and RT-PCR assays revealed that carnosol inhibited the activity and downregulation the expression of MMP-9. Mechanistically, we demonstrated that carnosol suppressed the activation of STAT3 signaling pathway through a ROS-dependent targeting of STAT3 to proteasome-degradation in breast cancer cells (MDA-MB-231, Hs578T, MCF-7, and T47D). We show that blockade of proteasome activity, by MG-132 and bortezomib, or ROS accumulation, by N-acetylcysteine (NAC), restored the level of STAT3 protein. In addition, using chick embryo tumor growth assay, we showed that carnosol significantly and markedly suppressed tumor growth and metastasis of breast cancer xenografts. To the best of our knowledge, this is the first report which shows that carnosol specifically targets signal transducer and activator of transcription 3 (STAT3) for proteasome degradation in breast cancer. Our study further provide evidence that carnosol may represent a promising therapeutic candidate that canmodulate breast cancer growth and metastasis.

## Introduction

Breast cancer still represents the most common cancers as well as one of the leading causes of world-wide cancer-related mortality; it accounted for 2.09 million cases and 627,000 cancer-related deaths in 2018 (1). TNBC represents a heterogeneous subtype of breast cancers that belongs mainly to the basal-like breast cancers and is associated with aggressive clinical conditions, where targeted therapies are currently limited (2). TNBC is a diagnosis of exclusion since those cells are known to lack the expression of estrogen receptor, progesterone receptor, and human epidermal growth factor receptor (3). TNBC is characterized by a high proliferative index, and is considered to be very aggressive. Clinically, TNBC is indicative of poor prognosis especially that is known to relapse very quickly compared to other types of breast cancer (4). Although breast cancer therapies have witnessed enormous developments in recent years, no highly efficient anti-cancer treatment has been discovered. This might be due to the lack of complete understanding of the etiological factors underlying carcinogenesis, local invasiveness and distant metastasis. Therefore, identification of novel drugs that targets TNBC are increasingly needed.

Remodeling and degradation of the extracellular matrix (ECM) and basement membranes by proteolytic enzymes are essential steps in invasion and metastasis. The role of matrix metalloproteinases (MMPs) have been well recognized in promoting tumor invasion and metastasis (5–7). In relevance to metastasis, MMPs directly breaks down ECM proteins and expose cryptic peptide epitopes, thereby promoting cellular invasion (8, 9). They can modify integrins and cell adhesion molecules, as well as proteolytically activate potent cytokines such as TGF-β. This results in the induction of epithelial-to-mesenchymal transition (EMT), an inclusive phenotypic alteration which enhances cell motility (9–11). Moreover, MMPs can release soluble factors that can assist in establishing a metastatic niche in distant organs, encouraging tumor cells colonization (10). Interestingly, in response to reciprocal paracrine stimulation, MMPs are made and released by both tumor cells themselves and cells in their environment.

One of the MMPs that is strongly correlated with metastatic breast cancer and is associated with poor prognosis is MMP9 (12, 13). Depletion of MMP9 has been shown to decrease cancer progression in multiple animal models of cancer (14). To this end, the study of MMP9 regulation and its molecular characterization is crucial for development of novel therapeutic approaches for the treatment of breast cancer. Accumulating evidence overwhelmingly documents that MMP9 expression is controlled by several growth factors and cytokines such as epidermal growth factor (15), fibroblast growth factor (16), and interleukin-6 (17) as well as by oncogenic proteins including Ras and Src (18, 19). Notably, many cancers frequently exhibit increased overexpression of these molecules. Contextually, these molecules often transmit signals via the STAT3. Hence, it is possible that STAT3 activation is a common signaling intermediate that leads to the overexpression of MMP9 in malignant tumors. This suggestion is supported by the fact that constitutively activated STAT3 has been documented in several types of tumors, including breast cancer (20).

Phytochemicals such as polyphenols, flavonoids, terpenes, and alkaloids have been receiving significant attention, due to their diverse pharmacological properties, including cytotoxic and cancer chemopreventive effects (21, 22). One of the purified compounds that gain a lot of increasing recent interest is carnosol. Carnosol is a phenolic diterpene isolated from culinary herbs that include oregano, sage, and rosemary (23, 24). Nowadays, the interest in carnosol is on the rise largely due to its health promoting properties such as anti-inflammatory (25), anti-oxidant (26), neuroprotective (27), anti-microbial (28), and anti-cancer properties (29–31).

Recently, our laboratory investigated the anti-cancer effect of carnosol against TNBC cells. We found that carnosol caused G2 phase block as well as induced apoptosis and autophagy in MDA-MB-231 breast cancer cells (30). However, whether carnosol has an effect on the invasiveness of these cells is still undefined. Here, we report the carnosol significantly inhibited migration and invasion of MDA-MB-231 cells as well as downregulated the activity and expression of MMP9 *in vitro*. Moreover, we show that carnosol inhibited the STAT3 pathway by targeting STAT3 to proteasomal degradation. Finally, we show that carnosol significantly inhibited tumor growth and metastasis of TNBC cells.

## Materials and Methods

### Cell Culture, Chemicals, and Antibodies

Human breast cancer cells MDA-MB-231(cat# 300275), MCF-7 (cat# 300273) and T47D (cat# 300353) were purchased from Cell Line Service (CLS)-GmbH. Hs578T (cat# HTB-126) were purchased from ATCC-USA. MDA-MB-231, Hs578T, and MCF-7 were cultured DMEM (Hyclone) and T47D in RPMI (Hyclone). All media were supplemented with 10% fetal bovine serum (Hyclone), 100 U./ml penicillin/streptomycin. All Cells were maintained at 37°C under a humidified atmosphere containing 5% CO_2_. Carnosol, N-acetylcystein (NAC) were obtained from Sigma Aldrich. 3-methyladenine (3 MA), chloroquine, Bortezomib, and MG-132 were from Cell Signaling. Antibodies to STAT3, phospho-STAT3 (Tyr705), and β-actin were from Santa Cruz Biotechnology.

### Wound Healing Assay

Wound healing assay was done to evaluate MDA-MB-231 cells migration. MDA-MB-231 cells were grown in six-well plates overnight until confluency. Three wound lines were made for each sample through the confluent monolayer using a sterile plastic 10 μL pipette tip. After cells were washed twice with PBS, cells were incubated at 37°C in fresh serum-containing DMEM with or without carnosol at indicated concentrations. Photographs of each wound width were captured by inverted microscope at X100 magnification (Nikon Ti-U, Nikon) at indicated times. Wound closure was determined by measuring the distance between the edges of the wound at time 0 and 5 h. The distance (D) migrated by the cells was calculated as follow: D = (Size of the wound at *t* = 0 h - size of the wound at *t* = 5 h).

### Cell Viability

Cell viability was performed by Muse Count and Viability Kit (Millipore, Hayward, CA, USA) following the manufacturer's protocols. This assay based on differentially staining of viable and dead cells based on their permeability to two DNA binding dyes. The viability was determined by Muse™ Cell Analyzer (Millipore).

### Matrigel Invasion Assays

The invasiveness of the MDA-MB-231 cells was evaluated using BD Matrigel Invasion Chamber (8-μm pore size; BD Biosciences, Bedfrord, MA, USA) according to manufacturer's instructions as previously described (32). MDA-MB-231 cells (1 ×10^5^) were suspended in 0.5 mL of media containing vehicle or the indicated concentrations of carnosol were seeded into the upper chambers of the system, while DMEM supplemented with 10% fetal bovine serum was placed into the bottom wells in the system as a chemo-attractant and then incubated at 37°C for 24 h. Non-penetrating cells were removed from the upper surface of the filter with a cotton swab. Cells that have penetrated through the matrigel to the lower surface of the chamber were fixed with 4% formaldehyde, stained with DAPI, and counted under a fluorescent microscope. For quantification, the assay was done in duplicates and repeated three times.

### Gelatin Zymography

MDA-MB-231 (2.5 ×10^6^) cells were grown in serum-free DMEM with or without carnosol (25 and 50 μM). After 24 h incubation, the conditioned medium was collected from culture and then concentrated. Total of 30 μg protein was resolved on non-reducing 10% polyacrylamide gels containing 0.1% gelatin. After electrophoresis, the gels were washed for 1 h in 2.5% (v/v) Triton X-100 to remove SDS and then incubated overnight at 37°C in 50 mM Tris-HCl (pH 7.5), 150 mM NaCl, 0.5 mM ZnCl_2_, and 10 mM CaCl_2_ to allow proteolysis of the gelatin substrate. 0.5% Coomassie brilliant blue R-250 (Bio-Rad, CA, USA) staining was used to reveal gelatin-clear bands corresponding to the MMPs activity. Densitometry was performed using ImageJ software and band density was normalized to the non-specific band staining on the gel. Results shown represent two independent experiments.

### Measurement of Matrix Metalloproteinase-9 by ELISA

Cells were seeded in 6-well plates in the presence of vehicle (DMSO) or carnosol for 24 h. The conditioned medium was collected and the levels of secreted MMP-9 were determined using immunoassay kit (Abcam, Cambridge, UK) according to the manufacturer's protocol. The proteins present in the conditioned media were concentrated using the Amicon Ultra-0.5 protein purification and concentration column (Millipore). Levels of MMP-9 were normalized to the total protein level in each sample. The assays were performed three times in triplicates. Data are presented as mean values ± SEM.

### RNA Extraction and RT-PCR

Total RNA from vehicle- or Carnosol-treated MDA-MB-231 cells were prepared using Trizol reagent as described by the manufacturer (Life Technologies, Inc.). RNA expression of MMP-9 was determined by using the Qiagen OneStep RT-PCR kit (Qiagen) according to manufacturer's instruction. Equal amounts of RNA (500 ng) were used as templates in each reaction. The sequences of specific primers were as follows: MMP-9 forward, 5′- TTGACAGCGACAAGAAGTGG-3′, and reverse, 5′- CCCTCAGTGAAGCGGTACAT-3′; MMP-2 forward, 5′-TCTCCTGACATTGACCTTGGC−3′, and reverse: 5′-CAAGGTGCTGGCTGAGTAGATC−3′; GAPDH forward, 5′-GGCCTCCAAGGAGTAAGACC−3′, and reverse: 5′- AGGGGTCTACATGGCAACTG-3′. The PCR products were separated by 1.5% agarose gel and visualized by ethidium bromide staining. Representative results from three independent experiments are shown.

### Whole Cell Extract and Western Blotting Analysis

MDA-MB-231 cells (1.8 ×10^6^) were seeded per 100 mm tissue culture dishes and incubated for 24 h. After each treatment, cells were washed twice with ice-cold PBS, scraped, collected by spinning down. The cell pellets were then lysed in RIPA buffer (Pierce) containing protease inhibitor cocktail (Roche) and phosphatase inhibitor (Roche) followed by incubation for 30 min on ice. Cell lysates were centrifuged at 14,000 rpm for 20 min at 4°C, and the supernatants were collected. Total protein concentration was quantified by BCA protein assay kit (Thermo Scientific) and the lysates were adjusted with lysis buffer. Aliquots of 25 μg of total cell lysate were loaded and resolved onto 6–15% SDS-PAGE. After electrophoresis, proteins were transferred from the gels to nitrocellulose membranes (Thermo Scientific) and blocked for 1 h at room temperature with 5% non-fat dry milk in TBST (TBS and 0.05% Tween 20). The membranes were immunoblotted with specific primary antibodies in blocking buffer overnight at 4°C and then with horseradish peroxidase-conjugated secondary antibodies against rabbit or mouse IgG. Immunoreactive protein bands were detected by ECL chemiluminescent substrate (Thermo-Scientific) and chemiluminescence was detected using the LiCOR C-DiGit blot.

### Chick Embryo Tumor Growth and Metastasis Assay

The chick embryo tumor growth assay was performed by INOVOTION (Société: 811310127), La Tronche-France as previously described (32). Fertilized White Leghorn eggs, obtained from the Société Française de Production Agricole (SFPA, St. Brieuc, France), were incubated at 38°C with 60% relative humidity for 10 days. According to the French legislation, no ethical approval is needed for scientific experimentations using oviparous embryos (decree n° 2013–118, February 1, 2013; art. R-214–88). Animal studies were performed under animal experimentation permit N° 381029 and B3851610001 to Jean Viallet (INOVOTION). At stage E10, the chorioallantoic membrane (CAM) was dropped by drilling a small hole though the eggshell into the air sac and a 1 cm^2^ window was cut in the eggshell above the CAM. Cultured MDA-MB-231-GFP were detached by trypsinization, washed with complete medium and suspended in serum free DMEM. A 50 μl inoculum of 1 ×10^6^ MDA-MB-231-GFP cells was added onto the CAM of each egg. Eggs were then randomized in 4 groups of 15 eggs (to get sufficient surviving embryos at the end of the experiments). One day later, tumors began to be detectable. They were then treated for 7 days, every 2 days (E11, E13, E15, E17), by dropping 100 μl of either carnosol (50 or 100 μM), colchicine (2 μM), or vehicle (0.02 % DMSO) in PBS onto the tumor. At E19 the upper portion of the CAM was removed, transferred in PBS and the tumors were then carefully cut away from normal CAM tissue and weighed. In parallel, a 1 cm^2^ portion of the lower CAM was collected to evaluate the number of nodules, containing GFP-expressing cells. The fluorescent nodules were visualized *in situ* using whole mounts of tissue fixed in 4% formaldehyde in PBS and flattened between a hollow glass slide and a thick coverslip. In order to number the nodule, a thorough and complete scan of the piece of the lower CAM was done using Leica Macrofluo fluorescent microscope equipped with GFP filter. Chick embryos were sacrificed by decapitation.

### Statistical Analysis

Data were presented as means ± S.E.M. Differences between groups were analyzed using a Student's I-test for paired or unpaired values. *P*- values of <0.05 were considered to be statistically significant. Unless otherwise stated, experiments were repeated at least 3 times.

## Results

### Carnosol Decreases Migration and Downregulates the Expression and the Activity of MMP-9 in MDA-MB-231 and Hs578T TNBC Cells

**Figure 1:**
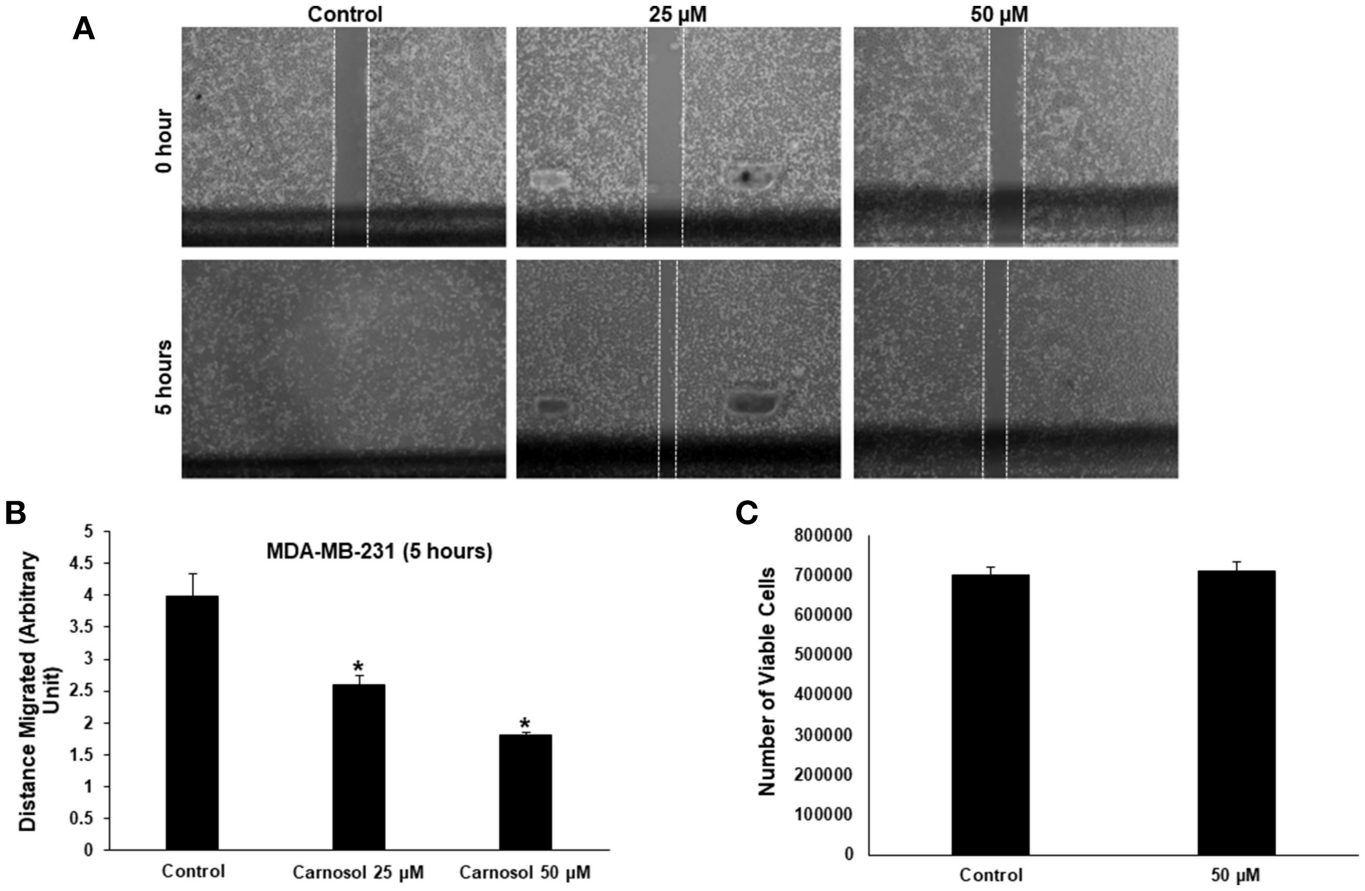
Carnosol inhibits the migration of MDA-MB-231 cells. **(A)** Confluent culture of MDA-MB-231 cells were wounded by scratching with a pipette tip and the cells were incubated in DMEM without and with the indicated concentrations of carnosol. The wound was measured with an inverted microscope x40 magnification and photographed. **(B)** Quantification analysis of the wound healing assay. Values represent the mean ± SEM (*n* = 3) distance (arbitrary unit) that the cells have migrated in 5 h (*p < *0.05*). **(C)** Cell viability of carnosol-treated MDA-MB-231 cells measured after 5 h treatment. Results shows that no significant difference in number of viable cells between carnosol-treated and control cells.

Especially in cancer cells of epithelial origin, cell migration represents a critical step in cell invasion. The effect of carnosol on the migratory capacityMDA-MB-231 and Hs578Tcells, was analyzed by wound-healing assay. Our results show that carnosol significantly inhibited cellular migration of MDA-MB-231 (Figures 1A,B) and Hs578T cells (Supplementary Figure 1).

To rule out that the inhibition of migration is due to drug-induced cell death, cells from control and carnosol-treated well were collected at the end of the experiment by trypsinization and the number of viable cells was measured. As is shown in Figure 1C, the number of viable cells did not decrease in carnosol-treated wells when compared to control.

### Carnosol Inhibits the Invasive Capacity of MDA-MB-231 Cells

**Figure 2:**
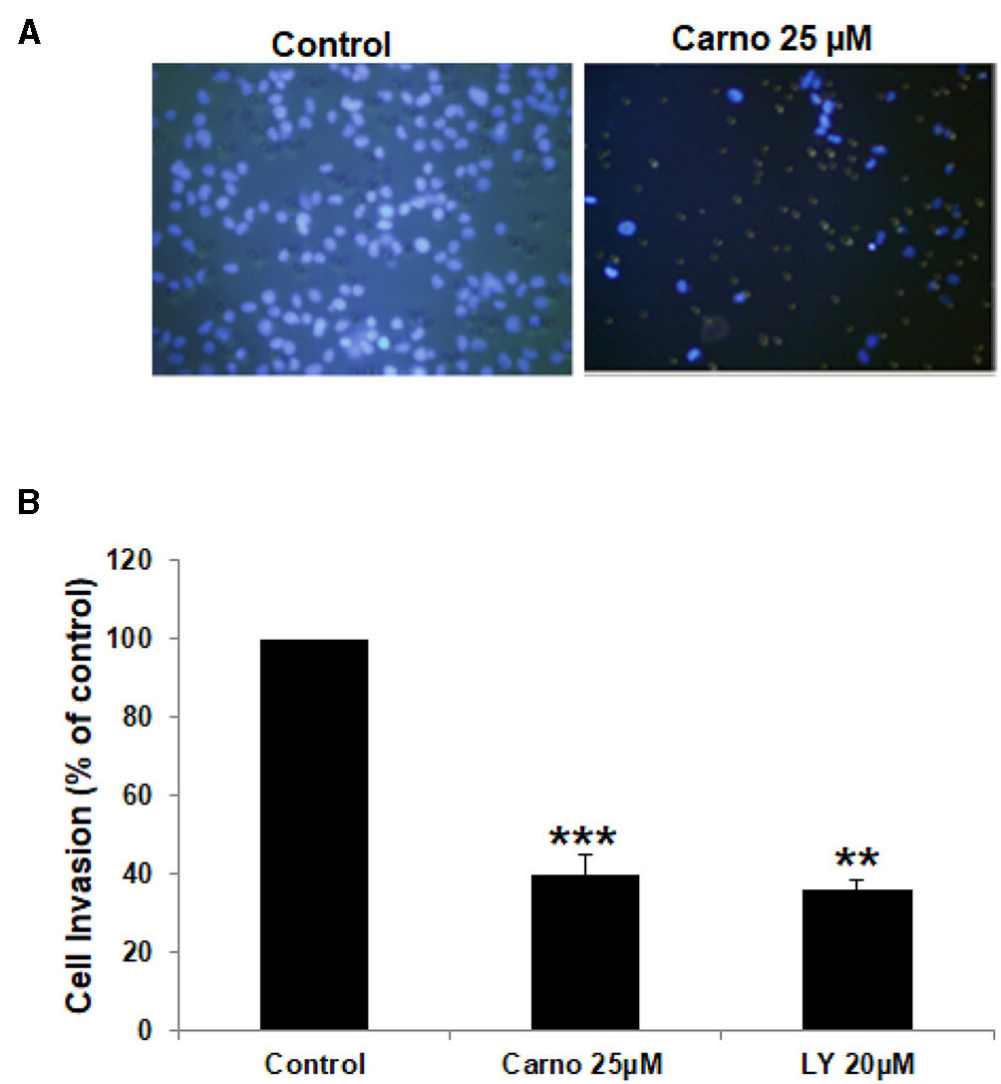
Carnosol inhibits the invasion capability of MDA-MB-231 cells. **(A)** MDA-MB-231 cells were incubated for 24 h with or without 25 μM carnosol and LY294002 (20 μM). Cells that invaded into the matrigel were scored as described in Materials and Methods. **(B)** Quantification of invaded MDA-MB-231 into the matrigel. Values represent means ± SEM, *n* = 3 (**p < 0.005 and ***p < 0.001).

Increased invasiveness is a hallmark of breast cancer metastasis. Having shown that carnosol significantly suppressed the migration ability of MDA-MB-231, we next sought to test the effect of carnosol on the invasive potential of MDA-MB-231 cells. We found that the invasive ability of MDA-MB-231 was significantly reduced by carnosol compared with the control cells. Carnosol treatment significantly reduced the number of cells invading the Matrigel coated membrane by 60% (Figure 2). Of note, LY294002, a drug used as positive control, strongly suppressed the invasion of MDA-MB-231 as well (Figure 2). These results indicate that carnosol has a significant suppressive capacity on the invasion of breast cancer cells.

### Carnosol Downregulates the Expression and the Activity of MMP-9 in MDA-MB-231 Breast Cancer Cells

**Figure 3:**
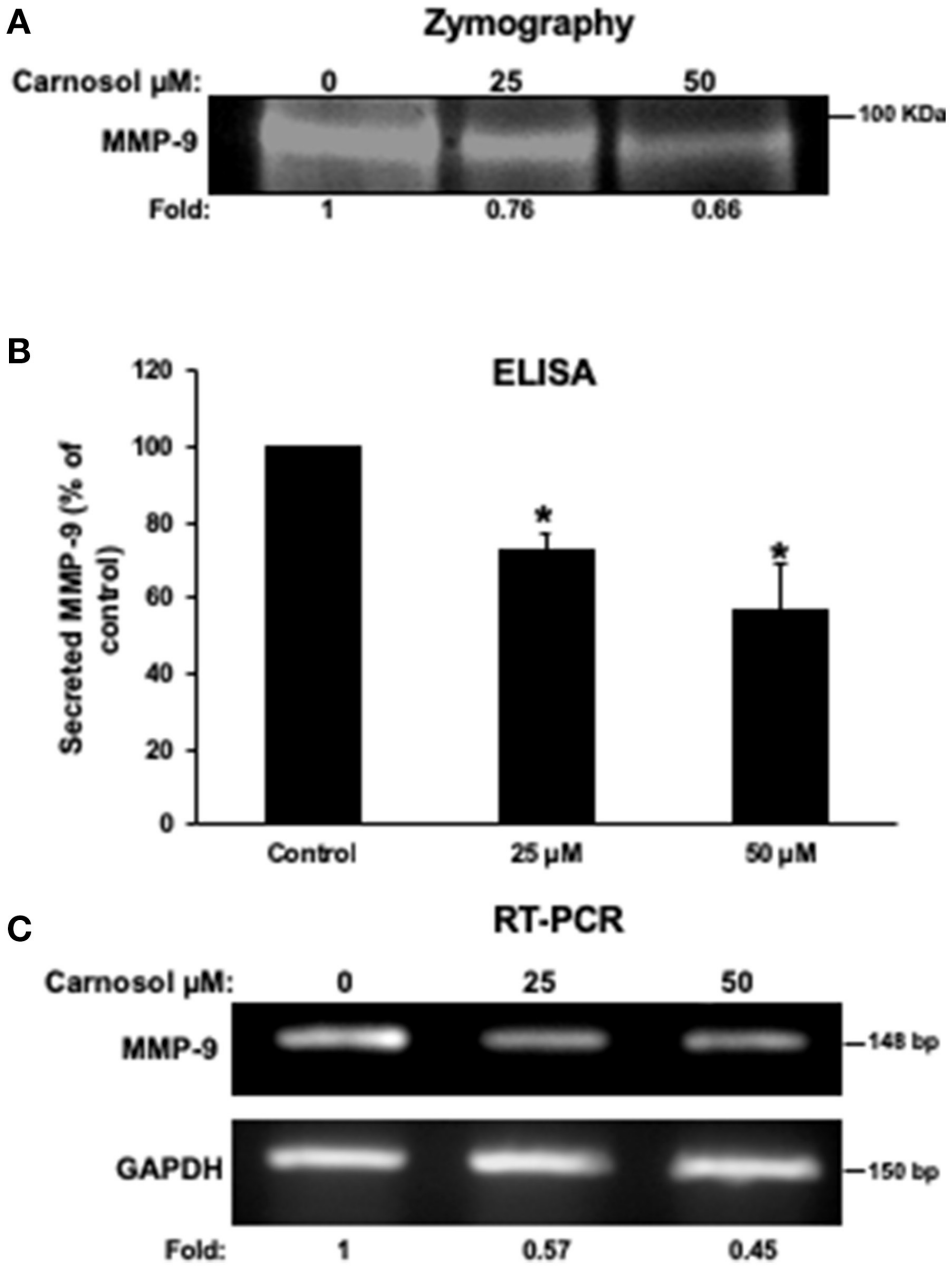
Carnosol reduces the activity and the expression of MMP-9 in MDA-MB-231 cells. **(A)** Activity of MMP-9 in carnosol-treated MDA-MB-231 cells. Cells were treated with 25 and 50 μM carnosol for 24 h and then subjected to gelatin zymography, to measure the activity of MMP-9. **(B)** Effects of carnosol on the secretion of MMP-9 in conditioned media. The levels of secreted MMP-9 were determined using immunoassay kits as described in Materials and Methods. Values represent the mean ± SEM (*n* = 3). **(C)** Effects of carnosol on the expression of MMP-9 mRNA. Cells were treated with vehicle (DMSO) or carnosol (25 and 50 μM) for 24 h and the subjected to RT-PCR to analyze the mRNA level of MMP-9 transcript. GAPDH was used as an internal control. Values represent the mean ± SEM (*n* = 3) (*p < 0.05).

It is well known that MMPs can degrade ECM to facilitate cancer cell migration and invasion. Hence, we analyzed the effect of carnosol on the activity of MMP-2 and MMP- in the conditioned media using gelatin zymography assay. As shown in Figure 3A, carnosol significantly reduced the activity of MMP-9 (Figure 3A) in a concentration-dependent manner. On the other hand, the activity of MMP-2 was unaffected by carnosol (Supplementary Figure 2A). Next, to test whether carnosol inhibits breast cancer cell invasion by affecting the expression of MMP-9, we examined the expression level of MMP-9 in the conditioned media. As shown in Figure 3B, levels of secreted MMP-9 were significantly reduced in carnosol-treated MDA-MB-231 cells. Further, RT-PCR analysis was performed to evaluate the mRNA expression of MMP-9. Our results revealed that MMP-9 expression was down-regulated in carnosol-treated cells (Figure 3C). The level of MMP-2 transcripts in carnosol-treated cells was comparable to control cells (Supplementary Figure 2B). Altogether, these data clearly indicate that carnosol suppresses the expression and activity of MMP-9 activity.

### Carnosol Inhibits STAT3 Pathway by Downregulating Both Active and Total STAT3 Specifically in Breast Cancer Cell Lines

STAT3 plays a crucial role in signaling pathways that promote cellular proliferation, tumor angiogenesis, invasion, and migration. The active form of STAT3 is constitutively and aberrantly activated in many types of cancers including TNBC, where it promotes tumorigenesis (33). Thus, STAT3 is widely recognized as a promising therapeutic target in cancer treatment.

**Figure 4:**
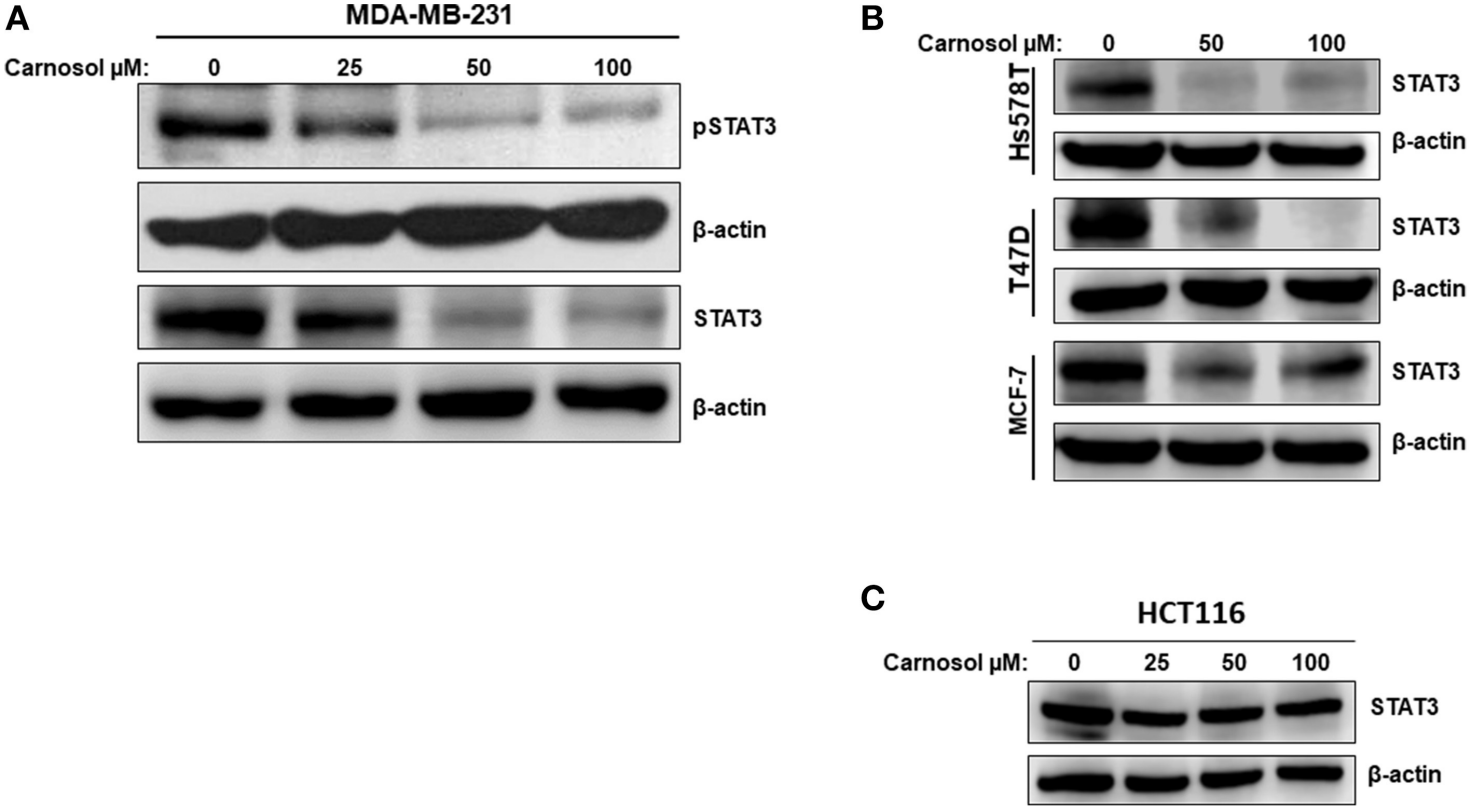
Carnosol inhibits the STAT3 signaling pathway. **(A)** Concentration-dependent decrease of phospho-STAT3 and STAT3 protein in carnosol-treated MDA-MB-231 cells. Cells were treated with vehicle (DMSO) or indicated concentrations of carnosol for 24 h, then whole-cell extracts were subjected to Western blot analysis for the phosphorylated and non-phosphorylated form of STAT3 and for β-actin (loading control). **(B)** Carnosol decreases the level of STAT3 protein in Hs578T, MCF-7, and T47D breast cancer cell lines. Whole cell lysates from different breast cancer cell lines treated with the indicated concentrations of carnosol were resolved on 8% SDS-PAGE and analyzed by western blot for STAT3 protein. **(C)** Western blot analysis of STAT3 protein level in HCT116 colorectal cancer cells. Western blotting was performed as described above.

Whether carnosol affects constitutively active STAT3 in MDA-MB-231 cells was investigated. Toward this, cells were with carnosol (25, 50, and 100 μM) for 24 h. We found that carnosol dramatically downregulated the level of phosphoSTAT3 in a concentration-dependent manner (Figure 4A, upper panel). Strikingly and unexpectedly, cells treated with carnosol showed a marked reduction in the level of total STAT3 (Figure 4A, lower panel). This finding prompted us to investigate whether carnosol inhibits STAT3 pathway in other breast cancer cell lines by downregulating the level of total STAT3. Toward this, the level of total STAT3 was assessed in three other breast cancer cell lines (Hs578T, MCF-7, and T74D) cell line. We found that carnosol also caused a marked decrease in the level of total STAT3 in these three cell lines as well (Figure 4B). Interestingly, and in agreement with previous work (31), we found that the expression of STAT3 was not affected in HCT116 colorectal cancer cells after carnosol treatment (Figure 4C). In summary, our data strongly suggests that carnosol inhibits STAT3 pathway through a breast cancer-specific mechanism.

### Carnosol Targets STAT3 to Proteasome-Mediated Degradation

**Figure 5:**
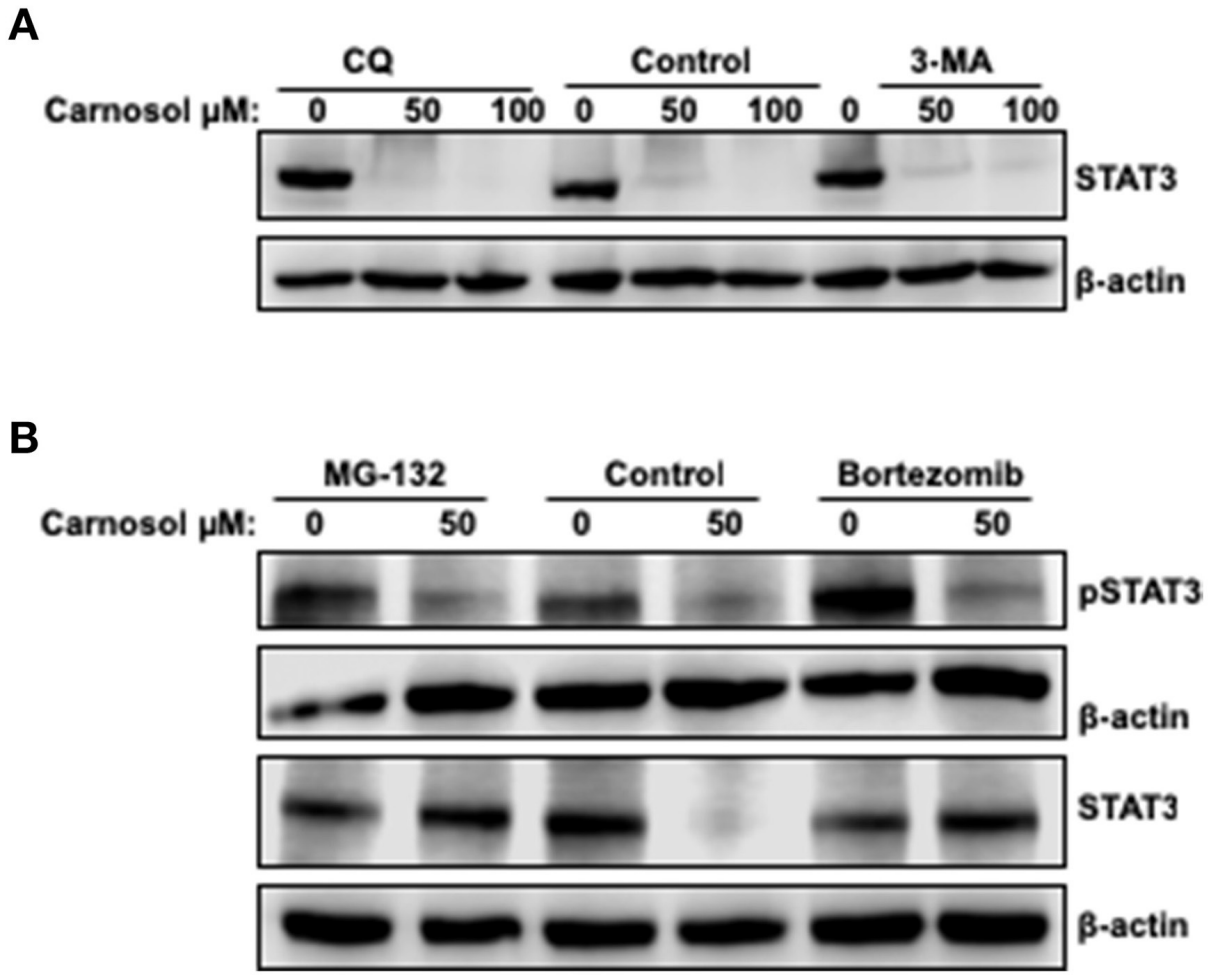
Proteasome-dependent degradation of STA3 protein in carnosol-treated MDA-MB-231 cells. **(A)** Western blot analysis of STAT3 protein level in MDA-MB-231 cells pre-treated with the autophagy inhibitors 3-MA and CQ. Cells were pretreated with or without 3-MA (50 mM) and CQ (50 μM) for 1 h and then carnosol was added, and cells were further incubated for 24 h. **(B)** Carnosol induces proteasome-dependent degradation of STAT3. Inhibitors of the proteasomes (MG-132 and Bortezomib) restored STAT3 protein to a level comparable to control cells. MDA-MB-231 were pre-treated for 1 h with or without MG-132 (15 μM) or Bortezomib (25 nM) before treatment with carnosol at the indicated concentrations. Whole cells lysate were resolved on 8% SDS-PAGE and analyzed by Western blot for STAT3 and phospho-STAT3 protein.

To investigate the mechanism by which carnosol downregulates the level of total STAT3 in breast cancer cells, the level of STAT3 mRNA transcript in control and carnosol-treated MDA-MB-231 cells was examined by qRT-PCR. Our results showed no significant changes in mRNA levels between control and carnosol-treated cells (data not shown) suggesting that downregulation of STAT3 occurred post-transcriptionally. We have previously shown that carnosol triggered autophagy in breast cancer cells (30). We therefore decided to examine whether STAT3 was degraded by autophagy. We found that, blockade of early stage autophagy (autophagosome formation) by 3-MA and late stage autophagy (autophagolysosome formation) by CQ did not restore the level of STAT3 protein (Figure 5A). This suggests that the decrease of STAT3 proteins in response to carnosol is autophagy-independent.

To determine whether proteasomal activity is involved in carnosol-mediated decrease of STAT3 protein, we first pre-treated MDA-MB-231 cells with or without the proteasome inhibitors MG-132 and bortezomib for 1 h and then with carnosol. As shown in Figure 5B, both inhibitors efficiently restored STAT3 to a level comparable to that in the control untreated cells. This is a clear indication that carnosol targets STAT3 to proteasome degradation. In addition, we found that restoration of STAT3 protein did not restore the level of phosphorylated STAT3 (Figure 5B). We have also examined the effect of proteasome inhibitor on STAT3 in another TNBC cell line, namely Hs578T. As shown in Supplementary Figure 3, bortezomib restored the level of STAT3 protein. Altogether, our data suggest that carnosol might exert its effect, at least partly, through inhibition of STAT3 signaling pathway by targeting STAT3 to proteasome degradation in breast cancer.

### Carnosol Promotes Proteasome Degradation of STAT3 Through a ROS-Dependent Mechanism

**Figure 6:**
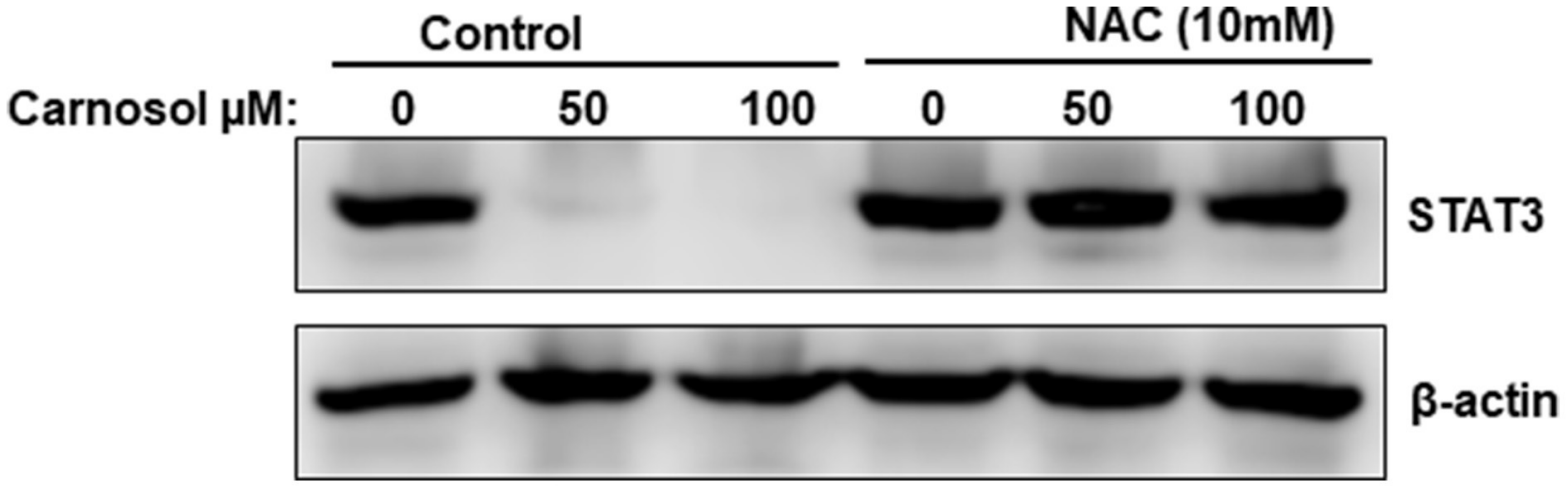
ROS-dependent proteasome degradation of STAT3. MDA-MB-231 were pre-treated for 1 h with the ROS scavenger, NAC, before adding carnosol (50 and 100 μM). Whole cells lysate were resolved on 8% SDS-PAGE and analyzed by Western blot for STAT3 protein.

We and others have previously showed that carnosol induced ROS in breast (30) and colon (31) cancer cells. This prompted us to test whether ROS contribute to STAT3 degradation. We pre-treated MDA-MB-231 cells with the ROS scavenger, NAC for 1 h, and this was followed by treatment with or without carnosol. As shown in Figure 6, blockade of ROS efficiently rescued STAT3 from proteasomal degradation. Similar results were obtained with Hs578T cells (Supplementary Figure 4). This result demonstrates that carnosol targets STAT3 to proteasome degradation through a ROS-dependent mechanism.

### Carnosol Inhibits Tumor Growth and Metastasis of MDA-MB-231 Cells

**Figure 7:**
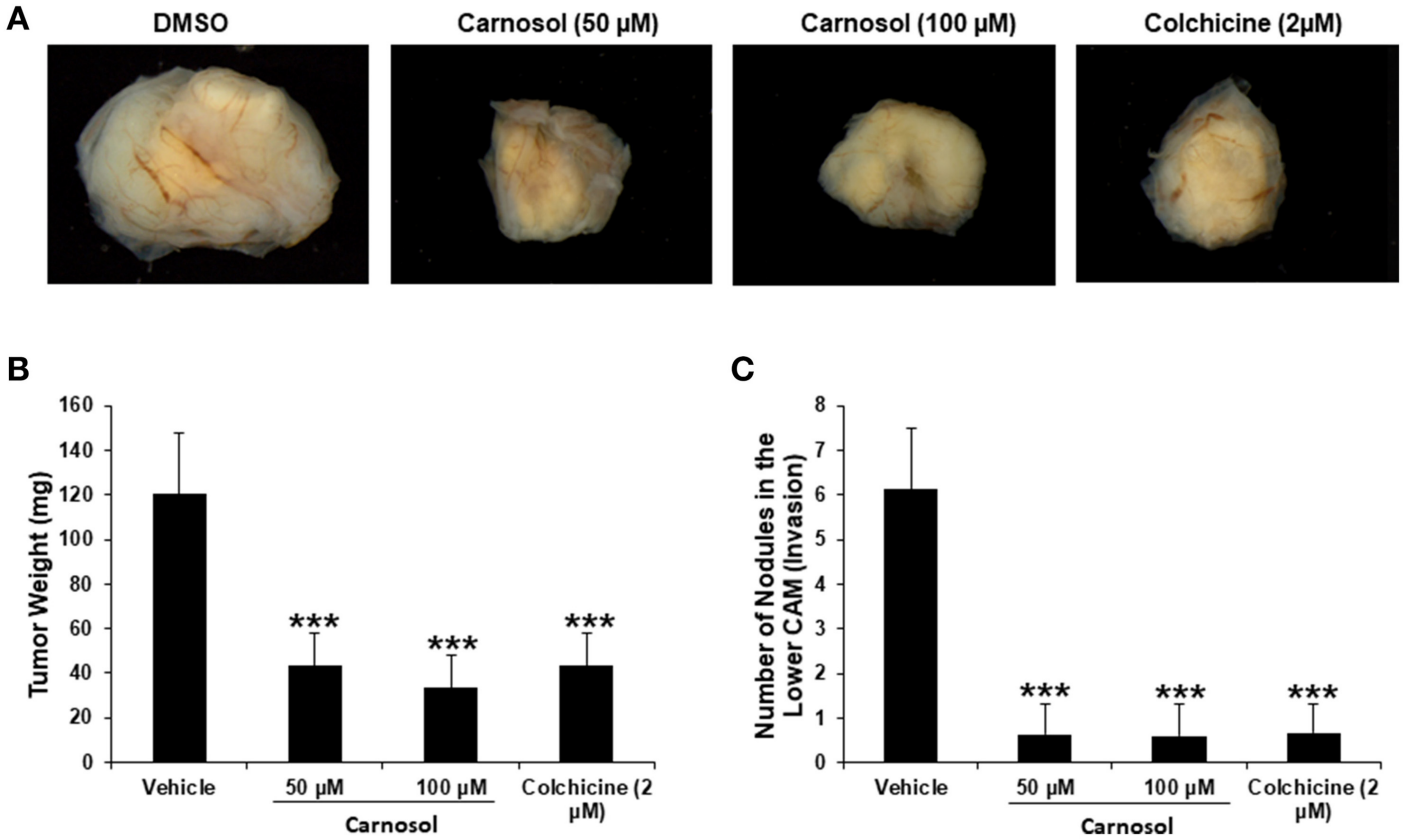
Anti-tumor growth and anti-metastatic activity of carnosol on breast tumor in chick embryo chorioallantoic membrane assay **(A)** MDA-MB-231 (1 ×10^6^) cells were grafted on the CAM of 10 day (E10) chick embryo. Tumors were treated every 48 h with carnosol (50 and 100 μM) as described in Materials and Methods. At E19, tumors were collected and weighted. **(B)** Quantification of tumor weight in vehicle-, colchicine- and carnosol-treated chick embryo. **(C)** Anti-metastatic effect of carnosol. Quantification of nodules observed in the lower CAM of chick embryo treated with vehicle, colchicine or indicated concentrations of carnosol. Columns represents mean; bars represent SEM (***p < 0.001).

We recently showed that carnosol significantly decreased the viability and colony growth of MDA-MB-231 cells (30). To further confirm these *in vitro* activities of carnosol, we investigated its effect on tumor growth *in vivo*, using the chick embryo model. We implanted highly invasive MDA-MB-231 cells on the chorioallantoic membrane (CAM) and then we treated the formed tumors with vehicle (DMSO), colchicine or carnosol. At E 19, we recovered the tumors from the upper CAM and weighed each. Our results demonstrated that carnosol at concentrations of 50 and 100 μM significantly inhibited tumor growth by 65 and 75%, respectively, compared with the DMSO treatment (Figures 7A,B). Colchicine treatment (2 μM) reduced tumor growth by 65%. Furthermore, we examined whether carnosol has the ability to inhibit metastasis *in vivo*. To this end, we counted the number of nodules in the lower CAM in DMSO-, colchicine- and carnosol-treated tumors. We found an average of 6 nodules in the lower CAM of vehicle-treated chick embryo, while an average of only 0.7 nodules were counted in carnosol-treated embryos (Figure 7C). Taken together, our data demonstrates that carnosol strongly inhibits breast tumor growth and metastasis *in vivo*. Of note, carnosol showed no cytotoxicity, as there was no difference in the number of surviving embryos in control and carnosol-treated embryo (data not shown).

## Discussion

The aim of this study was to investigate the efficacy of carnosol to inhibit breast cancer tumor growth and metastasis *in vivo*. We report that carnosol markedly reduced, *in vivo*, the growth of tumor derived from MDA-MB-231 cells and significantly inhibited invasion and metastasis both *in vitro* and *in vivo*. We demonstrated that carnosol exerts its effect against breast cancer, at least partly, through downregulation of the activity and the expression of MMP-9, inhibition of STAT3 signaling pathway through a ROS-dependent proteasome degradation of STAT3 protein.

Cancer metastasis is a complex process involving several steps, a critical one of which is invasion, which involves proteolytic degradation of the ECM. It has been reported that elevated levels of MMPs are linked to aggressiveness of breast cancer and metastatic potential (34). Inhibiting the expression or activity of these proteases is considered a potential therapeutic approach against breast cancer. Here, we showed that the anti-metastatic activity of carnosol involves suppressing both the activity and expression of MMP-9. This implies that carnosol is expected to reduce the degradation of the ECM.

Several signaling pathways, including STAT3 are known to regulate the expression of various genes involved in the process of tumor migration and invasion. By virtue of its ability to upregulate MMP-9, STAT3 appears to robustly enhance tumor migration and invasion of MDA-MB-231 cells. Relevantly, a recent study by Kim and collaborators showed that suppression of STAT3 phosphorylation by LYR71, a derivative of trimeric resveratrol, was associated with an inhibition of MMP-9 in MDA-MB-231 cells (35). Moreover, MMP-9 activation required the recruitment of STAT3 to MMP-9 promoter and that LYR71 reduced MMP-9 transcripts by blocking STAT3 on the MMP-9 promoter (36).

Song et al., showed that juxtaposed Stat3/AP-1 element on the MMP-9 promoter plays a crucial role in the manner of enhancer in the activation of MMP-9 gene. Indeed, they showed that the functional cooperation of the Stat3 and AP-1 transcription factors is required for the transcription of MMP-9 gene in breast cancer cells (37). It is also noteworthy to mention that, in addition to its role in migration, invasion and metastasis, MMP-9 is known to play a crucial role angiogenesis as well as tumor formation (38). Most relevantly, Dechow et al. showed that the expression of MMP-9 is induced by STAT3 (39). Indeed, overexpression of constitutively active form of Stat3c in breast cells led to a significant upregulation (up to 4-fold increase) of MMP-9 mRNA transcript. Added to that, these authors also showed that MMP-9 expression correlates with that of activated Stat3 in human breast cancer specimens. This is in accordance with other recent report showing that inhibition of STAT-3 abolishes the activity of MMP-9 in MDA-MB-231 cells (40). Finally, Knock down of STAT3 in the multidrug resistant breast cancer, SK-BR-3/EPR, cell line inhibited cell invasion and downregulated MMP-9 in these cells (41). Interestingly, here we showed that carnosol dramatically reduced the level of STAT3, a transcription factor required for MMP-9 expression, and downregulated MMP-9 in MDA-MB-231 cells. We hypothesize that depletion of STAT3, by carnosol, contribute, although may be not solely, to the inhibition of transcription of MMP-9 gene which results in the downregulation of MMP-9 protein/activity.

Persistent activation of STAT3 has been described in several cancers including breast cancer. A crucial role of STAT3 in tumor onset and progression, tumor cell invasion, metastasis and angiogenesis has been largely demonstrated. Therefore, targeting STAT3 is an attractive approach given that it assaults cancer on multiple fronts (36). Recently, carnosol was shown to attenuate activation of STAT3 signaling by inhibiting its phosphorylation, while having no effect on the inactive STAT3 in HCT116 human colon cancer cells (31). In agreement with this finding, we also found that carnosol inactivated STAT3 signaling pathway in a panel of breast cancer cell lines (MDA-MB-231, Hs578T, MCF-7, and T47D). Strikingly, here we demonstrated that inactivation of STAT3 signaling in all breast cancer cell lines tested involved a different mechanism. Indeed, targeting of total STAT3 protein to proteasomal degradation was notable, hence suggesting that inhibition of STAT3 by carnosol involves a cancer-type specific mechanism(s). In recent years, several potent and selective inhibitors of STAT3 of synthetic or natural origin were described. These inhibitors appear to act via a direct or indirect mode of action. While indirect inhibitors block the upstream effectors regulating STAT3 activation, direct inhibitors, on the other hand, block phosphorylation, dimerization, nuclear translocation, and DNA binding of STAT3 (42, 43). Recent studies showed that paeoniflorin (PF), a natural compound, suppressed growth, migration, invasion and tumor growth of U87, U251, T98G glioblastoma cells. Interestingly, PF was shown to target STAT3 to proteasome degradation (44, 45). However, the exact mechanism through which PF targets STAT3 to proteasome degradation remain unknown. Similar to PF, we found that carnosol also inhibits STAT3 signaling through a mechanism involving a ROS-dependent proteasome degradation of STAT3 protein. We have previously shown that carnosol induced ROS generation in a concentration-dependent manner in MDA-MB-231 cells. Here, we showed that inhibition of ROS accumulation by NAC also restored the level of STAT3 protein. Our results strongly suggest that carnosol-mediated accumulation of ROS, through a yet to discover mechanism, targets STAT3 to proteasome degradation. This contributes, at least partly, to the inhibition of cell migration, invasion, metastasis and tumor growth of breast cancer. Further studies are needed to demonstrate the effect of carnosol on STAT3 in tumors themselves. To the best of our knowledge, carnosol is the first compound reported to specifically target STAT3 for proteasome degradation in breast cancer.

A large body of evidence supports the notion that ROS can also inhibit metastasis of breast cancer. Baicalin, for example, a natural flavonoid, was shown to suppress migration/invasion of MDA-MB-231 cells via a ROS-mediated activation of p38/JNK signaling pathway (46). Also, theaflavins, natural polyphenols, were shown to inhibit migration and downregulate levels of MMP2 and MMP9 in MDA-MB-231 through a mechanism involving ROS generation (47). Likewise, phenethyl isothiocyanate (PEITC) and benzyl isothiocyanate (BITC), two phytochemicals, were shown to inhibit migration and invasion of human non-small cell lung cancer cells through a ROS-dependent mechanism (48). Based on our findings, we hypothesize that ROS might contribute, albeit not solely, to the carnosol-mediated inhibition of cellular migration, invasion and tumor growth of TNBC through proteasomal degradation of STAT3. Thus, this work along with our previous findings (30) further suggests that carnosol may serve as a novel and effective anticancer agent for treatment of the highly invasive and metastatic TNBC.

Finally, it is noteworthy to mention that large number of experiments carried out in animals showed that carnosol is safe. Here, we showed that carnosol at concentrations of 50 and 100 μM has no toxicity to chick embryo. In addition, toxicological effects of carnosol performed in C57BL mice treated with a dose of as high as 200 mg/kg for five successive days also failed to show any potential toxic effects (49). Moreover, a study in using Sprague-Dawley rats showed that 1% of carnosol supplemented diet (AIN-76A) had no overt side effects on body weight (50). Toxicological studies of carnosol-enriched rosemary extract at high doses ranging from 180 to 400 mg/kg/BW/day have been conducted in male and female rats with no observable side effects (51). Finally, FDA has given “Generally Recognized As Safe” status for carnosol-enriched rosemary extract herbal drug formulations (52). Similarly, the European Commission has also approved some of the standardized rosemary extracts with similar potency to that of carnosol in food additives (52).

**Figure 8:**
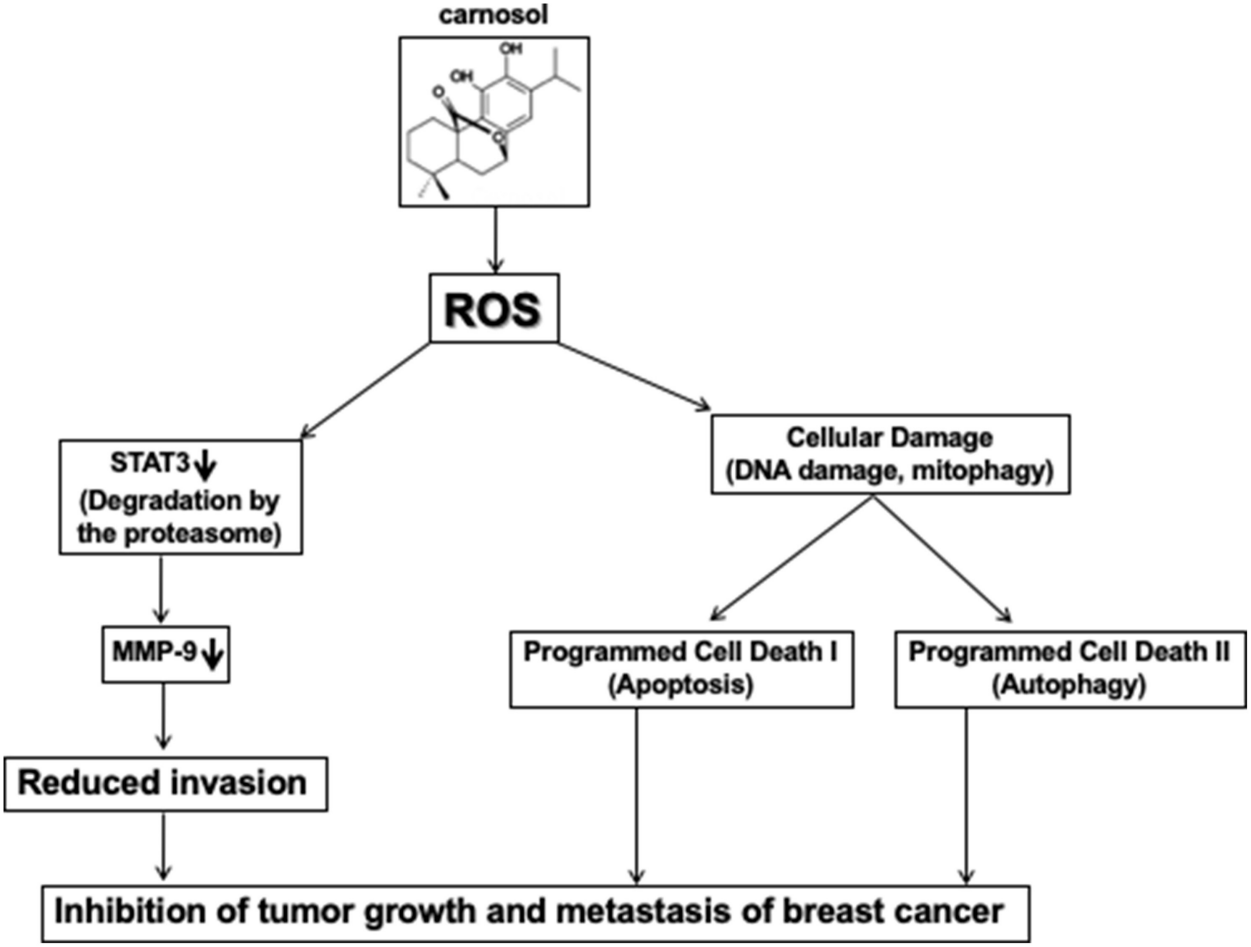
Hypothetic model illustrating the mechanism of action of carnosol in inhibiting tumor growth and metastasis of breast cancer.

In conclusion and based on our previous findings (30), and this present work, we hypothesized a schematic model shown in Figure 8, presenting possible mechanisms for the inhibitory effect of carnosol on cell migration, invasion, tumor metastasis and tumor growth of TNBC cells.

## Data Availability

The datasets generated for this study are available on request to the corresponding author.

## Author Contributions

HA, HE, and AE performed the migration assay and all Western blots. YA performed gelatin zymography and MMP9 analysis. SA performed the invasion assay. RI designed the project, analyzed the data, and wrote the manuscript. All authors reviewed the manuscript.

### Conflict of Interest Statement

The authors declare that the research was conducted in the absence of any commercial or financial relationships that could be construed as a potential conflict of interest.
