# Corrigendum: Carnosol, a natural polyphenol, inhibits migration, metastasis, and tumor growth of breast cancer via a ROS-dependent proteasome degradation of STAT3

**DOI:** 10.3389/fonc.2023.1235201

**Published:** 2024-02-29

**Authors:** Halima Alsamri, Hussain El Hasasna, Yusra Al Dhaheri, Ali H. Eid, Samir Attoub, Rabah Iratni

**Affiliations:** ^1^ Department of Biology, College of Science, United Arab Emirates University, Al Ain, United Arab Emirates; ^2^ Department of Pharmacology and Toxicology, Faculty of Medicine, American University of Beirut, Beirut, Lebanon; ^3^ Department of Pharmacology and Therapeutics, College of Medicine and Health Sciences, United Arab Emirates University, Al-Ain, United Arab Emirates

**Keywords:** triple negative breast cancer (TNBC), stat3, reactive oxygen species, proteasome, tumor growth, metastasis

In the published article, there was an error in **Figure 2** as published. The control image in **Figure 2A** (left panel) was previously published for a different drug as the experiments were performed in parallel and shared the same control. To eliminate any potential copyright issues, we have chosen to replace the control image in **Figure 2A** (control, left panel, blue square) with a new picture taken from a different field. The corrected **Figure 2** and its caption appear below.

**Figure 2:**
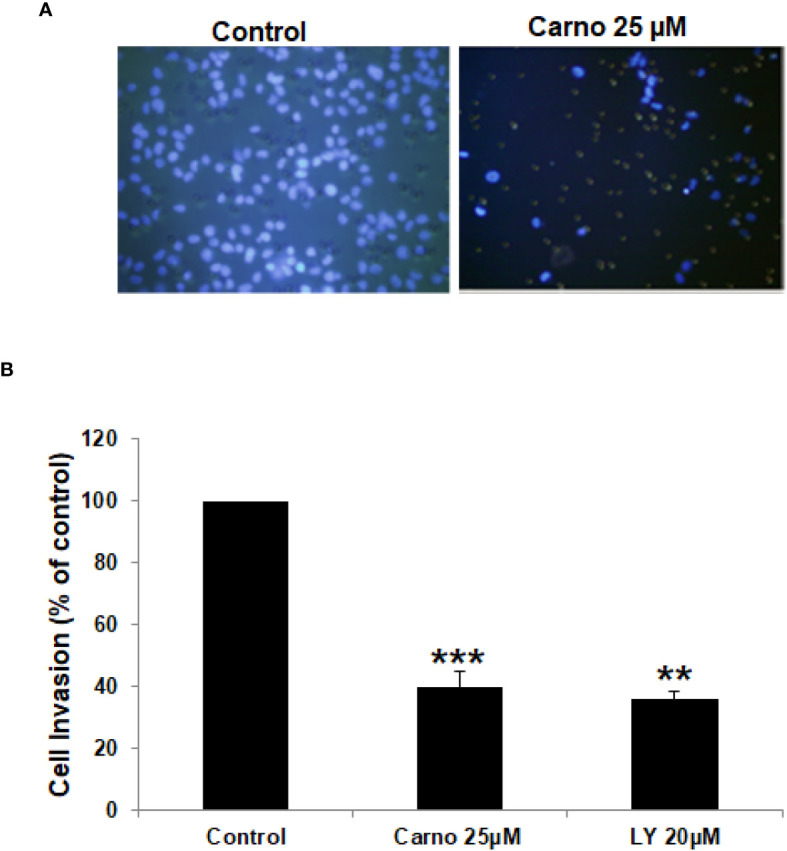
Carnosol inhibits the invasion capability of MDA-MB-231 cells. **(A)** MDA-MB-231 cells were incubated for 24 h with or without 25μM carnosol and LY294002 (20μM). Cells that invaded into the matrigel were scored as described in Materials and Methods. **(B)** Quantification of invaded MDA-MB-231 into the matrigel. Values represent means ± SEM, n = 3 (**p < 0.005 and ***p < 0.001).

The authors apologize for this error and state that this does not change the scientific conclusions of the article in any way. The original article has been updated.

