# Supplementary material for: Carnosol, a Natural Polyphenol, Inhibits Migration, Metastasis, and Tumor Growth of Breast Cancer via a ROS-Dependent Proteasome Degradation of STAT3
Source: Front Oncol. 2019 Aug 8;9:743. doi: 10.3389/fonc.2019.00743 (PMC6698796; doi:10.3389/fonc.2019.00743)
Supplement: Supplementary file 1 [file Data_Sheet_1.PDF]

Supplementary Material

Carnosol, a natural polyphenol, inhibits migration, metastasis and tumor growth of breast cancer via a ROS-dependent proteasome degradation of STAT3

Halima Al Samri[1], Hussain El Hasasna[1#], Yusra Al Dhaheri[1], Ali H. Eid[2], Samir Attoub[3] and Rabah Iratni[1*]

[1]*Department of Biology, College of Science, United Arab Emirates University, Al Ain, 15551, United Arab Emirates*

[2]*Department of Pharmacology and Toxicology, Faculty of Medicine, American University of Beirut, Beirut, Lebanon*

[3]*Department of Pharmacology & Therapeutics, College of Medicine & Health Sciences, United Arab Emirates University, Al-Ain, United Arab Emirates*

[#]Present Address: Cancer Cluster, College of Medicine, University of Saskatchewan, Saskatoon, S7N 5E5, Canada

*To whom Correspondence should be addressed:
R. Iratni, Department of Biology, United Arab Emirates University, P.O. Box: 15551, Al Ain, United Arab Emirates. Phone: +971 3 713 6526, Fax: +971 3 7134927, Email: R_iratni@uaeu.ac.ae

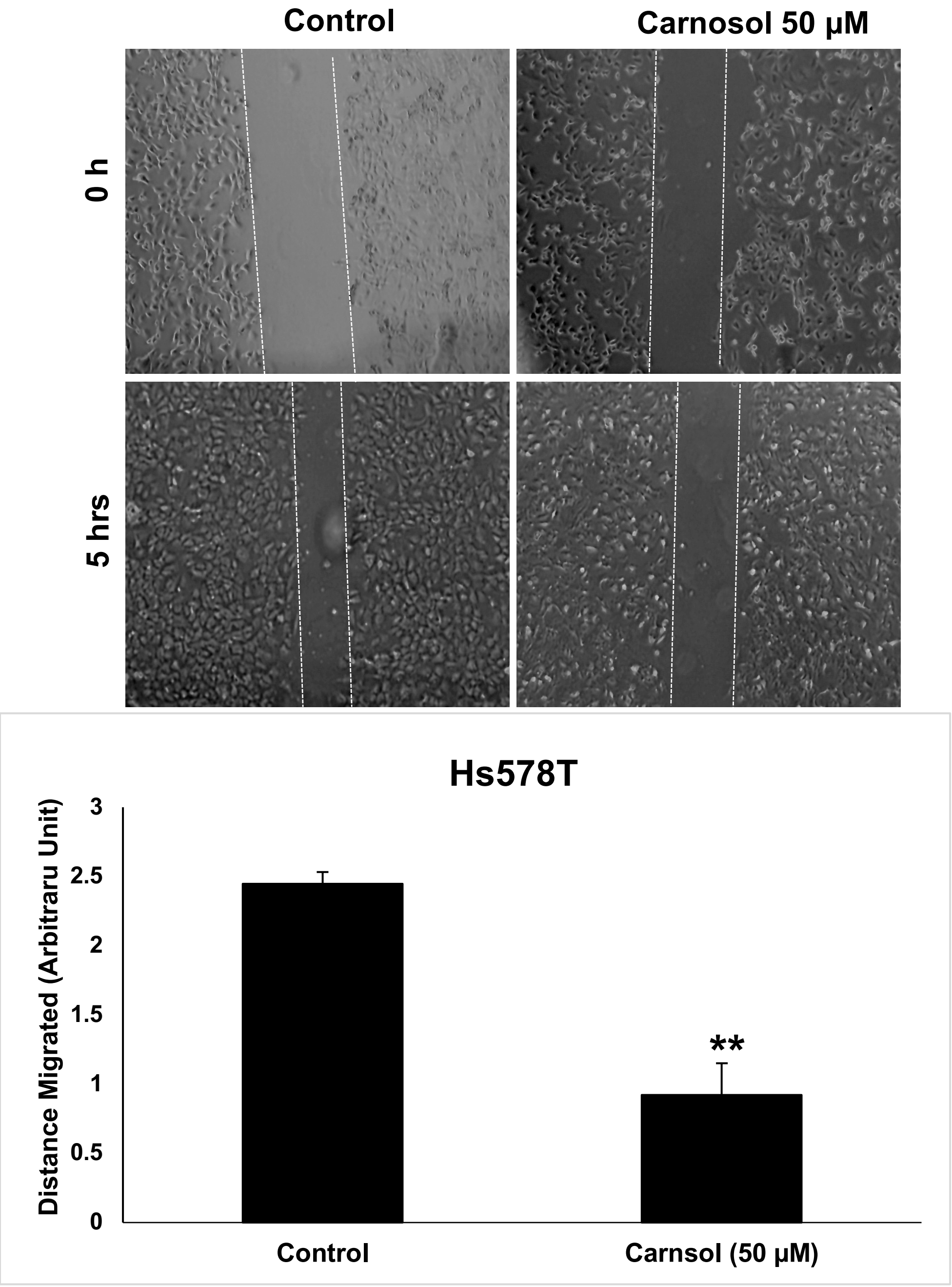

Supplementary Figure 1: Carnosol inhibits the migration of Hs578T cells. **(A)** Confluent culture of Hs578T cells were wounded by scratching with a pipette tip and the cells were incubated in DMEM without and with the indicated concentrations of carnosol. The wound was measured with an inverted microscope x 40 magnification and photographed. **(B)** Quantification analysis of the wound healing assay. Values represent the mean ± SEM (n=2) distance (arbitrary unit) that the cells have migrated in 5 h (***p<0.005*).

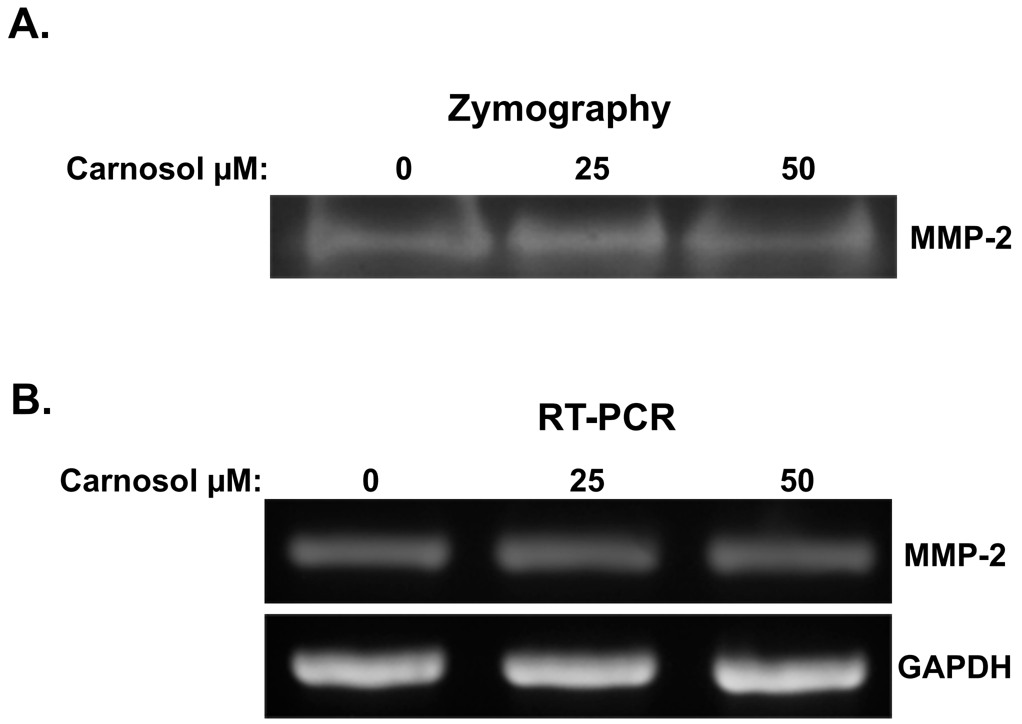

Supplementary Figure 2: Carnosol does not affect the expression nor the activity of MMP-2. **(A)** Activity of MMP-2 in carnosol-treated MDA-MB-231 cells. Cells were treated with 25 and 50 µM carnosol for 24 hours and then subjected to gelatin zymography, to measure the activity of MMP-2. **(B)** (**C**) Effects of carnosol on the expression of MMP-2 mRNA. Cells were treated with vehicle (DMSO) or carnosol (25 and 50 µM) for 24 hours and the subjected to RT-PCR to analyze the mRNA level of MM2-9 transcript. GAPDH was used as an internal control.

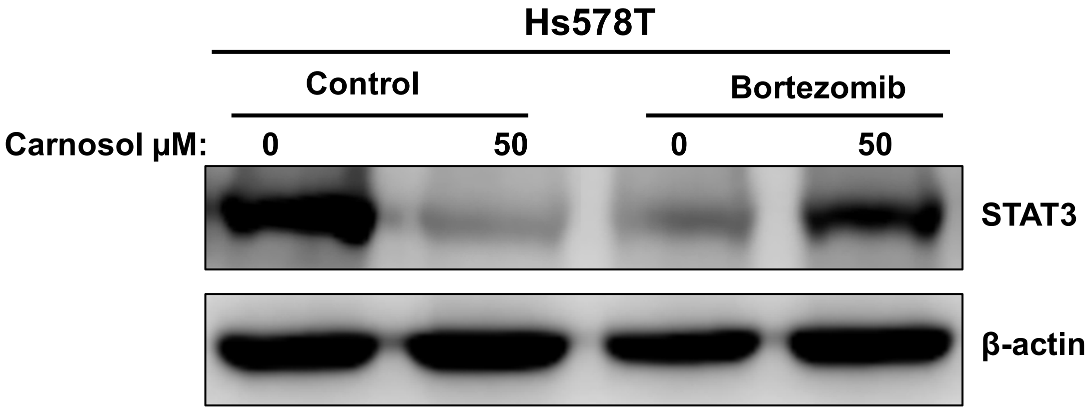

Supplementary Figure 3: Carnosol induces proteasome-dependent degradation of STAT3 in Hs578T. Inhibitors of the proteasomes (Bortezomib) rescued STAT3 protein from proteasome degradation. Hs578T cells were pre-treated for 1 hour with or without Bortezomib (25 nM) before treatment with carnosol at the indicated concentrations. Whole cells lysates were resolved on 8% SDS-PAGE and analyzed by Western blot for STAT3 protein.

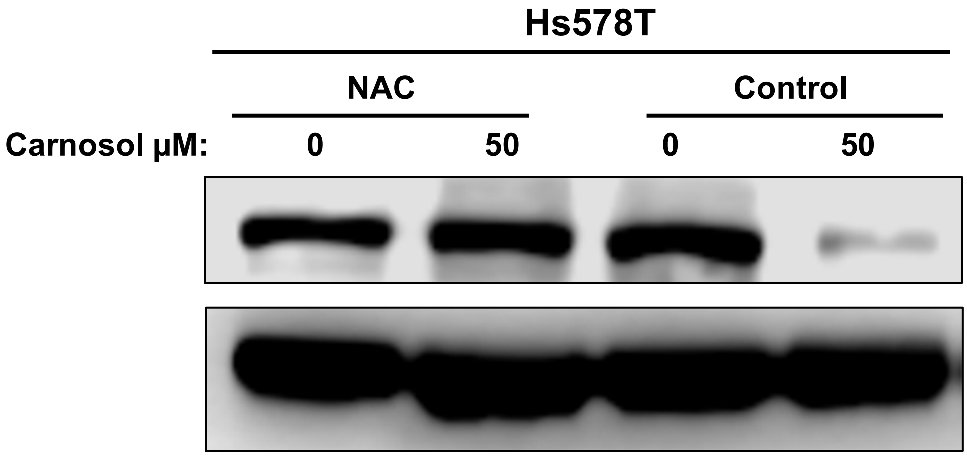

Supplementary Figure 4: ROS-dependent proteasome degradation of STAT3 in Hs578T cells. MDA-MB-231 were pre-treated for 1 hour with the ROS scavenger, NAC, before adding carnosol. Whole cells lysates were resolved on 8% SDS-PAGE and analyzed by Western blot for STAT3 protein.
